# An Address, Delivered at the Opening of the Dental College of Ohio, at Request of Faculty

**Published:** 1846-03

**Authors:** B. P. Aydelott

**Affiliations:** President of the Board of Trustees.


THE AMERICAN

### JOURNAL OF DENTAL SCIENCE.

Vol. VI.] MARCH, 1846. [No. 3.

### ARTICLE I.

*An Address, delivered at the opening of the Dental College of Ohio, at request of Faculty, by* Rev. B. P. Aydelott, D. D., President of the Board of Trustees.

What is the true measure of honor? Not birth, not wealth, no particular calling or pursuit in life, not high station, not human applause, not even great mental endowments—not one, not all of those things combined, to which the world too commonly bows down in homage. Some of these things are casual, and others of them may be very improperly obtained.

What, then, is the true measure of honor? We answer, without fear of successful contradiction, it is *usefulness—intelligent, voluntary usefulness.* This requires effort, and, for its highest achievement, demands much toil, much self-sacrifice. He who would be useful must lay himself out for it; he must plan, contrive and labor; he must overcome obstacles, nerve himself for every contest, and he must persevere. In no other way can he attain to much usefulness.

Since, then, usefulness is a matter of choice, and toil, and self-sacrifice, it must be the true measure of honor. No one can justly object to this test; all must feel it to be reasonable and right, though, unhappily, very few practically apply it either to themselves or to others.

With this standard of judgment, how meanly shall we think of much that is highly honored in the world; and how will it raise in our esteem, many of those classes and pursuits upon which a proud, unthinking world generally looks down with contempt. Were this standard universally set up, what a revolution in public sentiment would follow; what overturnings would take place! And we doubt not that such a day of righteous judgment will come. There is much in the world's progress to encourage the hope, that the time is drawing nigh, when all unreasonable, arbitrary and fictitious distinctions will be done away, and usefulness—in the most liberal, comprehensive and just sense of the term—usefulness will be universally regarded as the true measure of honor.

If the views now expressed are true and sound, are we not warranted in placing the medical profession, in all its departments, in the very first rank of honor? Let none object that this post must ever be conceded to the christian ministry. Certainly, they who devote themselves to this arduous and most responsible work are to be very "highly esteemed in love, for their work's sake." But it would be improper and unsafe to reckon the gospel ministry among worldly professions, and thus introduce an unseemly competition between it and others for precedence. The truth is, the ambassador of heaven can have no place in this world's ranks, just because his office must raise him above the highest, while his spirit ought to humble him to the feet of the lowest—make him cheerfully willing to be the servant of all.

But among earthly callings, we feel warranted in placing the healing art in the most elevated position. For what are the objects of all other pursuits? Are they not to minister to the support, the comfort, or the gratification of men, or to protect their rights? The agriculturist, the mechanic, the artist, the lawyer, the soldier, and the statesman can do no more than these. But suppose them all accomplished, what would they avail without life, without health? One touch of disease will stamp vanity on all this world's possessions, and mar its every enjoyment. But health will pour sunshine over the humblest condition, and sweeten even the coarsest morsel. We have somewhere read of a poor but laboring blacksmith; his eye was bright, his muscles firm, his step elastic. While on his way to dinner, and, as usual, humming a tune, or whistling in the gladness of overflowing health, he passed the door of a splendid mansion; the rich occupant of the dwelling long an invalid, could not but look with feelings of envy, upon the hale, happy smith, and calling him in, proposed to exchange conditions with him. The poor man eagerly, but thoughtlessly, assented. A few months reversed the scene. Idleness and luxury soon dimmed the eye, enfeebled the frame, and depressed the spirits of the once stalwart mechanic. This brought him to his senses. "Of what use," said he, "are this fine house, and costly furniture, and rich food? I have neither health nor appetite to enjoy them!" He would gladly have again exchanged conditions with his wiser friend, the now vigorous and happy blacksmith.

Health is the first of this earth's blessings, and the very basis and essential element of all its enjoyments:

> "Without the cheerful, active energy of health,
> No rapture swells the breast"
>
> *Armstrong, slightly altered.*

And so long as the human family is subject to weakness, pain and disease, the paramount usefulness of the healing art will be felt, and its practitioners will be entitled to proportionate honors.

But it is not our design to pronounce a eulogy on the medical profession. We wish merely to suggest a few words of counsel to those at whose request we stand here this day, and, through them, to all who have chosen the same department of the healing art. We say *department of the healing art*, because we believe that the rightful position of the dentist is not generally understood, and, consequently, his character not duly appreciated by the public. Even his fellow laborers do not, in this respect, always do him justice. His must be regarded as one branch of the healing art. He is, therefore, a medical practitioner. This is exactly his position; to this rank, and nothing less, is he most rightfully entitled. The science of medicine, it is well known, is so extensive, and requires such laborious study and long application to acquire an adequate knowledge of it, that, in every advanced community, its practitioners have, spontaneously, each for himself, chosen some particular part of the wide field, on which to lay out his mind and strength. Hence we have the physician, the surgeon, the accoucheur, the oculist, and, to name no more, the dentist. Each one of these professes to have devoted himself to the study and relief of a certain class of the great catalogue of human ills. The public have a right, therefore, to expect from him peculiar skill in this particular department. And we doubt not, but that, as medical science advances and enlarges its domain, and multiplies its objects of attention, it will be found absolutely necessary to increase these professional divisions. Instead of having some half a dozen different departments of the healing art, as we now have, we may have a score before the revolution of another century.

Permit us, here, briefly, to point out one good consequence which will be likely to result to the science and practice of dentistry, when every dentist regards himself, and is regarded by the public, as a member of the medical profession. He must, in this case, see more clearly the propriety and importance of going through a full course of medical study. The diseases of the mouth, about which he is specially concerned, must always sustain relations, more or less extensive, to other parts of the system and its different functions. Hence, no man can be a thoroughly qualified dentist who has not a general acquaintance with the human structure and its various processes, together with its morbid states and their appropriate remedies. In this way only, can he discern and duly appreciate the multitudinous and powerful, and often subtle influences, as cause and effect, between the diseases with which he has to do, and those affecting the whole or other parts of the system. Such relative knowledge will often throw a clear light upon what would otherwise be completely hidden from his view, and enable him successfully to treat what must otherwise baffle his utmost skill. When dentists generally are thus qualified, they cannot fail to assume their rightful rank, as professional men, before the public.

Such, then, being, in our view, the true position of the dentist—so useful, so honorable—we would respectfully, but briefly, call your attention to the principal duties which devolve upon him.

I. *Highly esteem and carefully cultivate your profession.*

It is too true, indeed, that selfishness is the main-spring of multitudes to exertion. And selfishness, it must be allowed, will do a great deal; but love for an object will do a vast deal more. He whose heart is in his profession, will pursue it with an ardor and an energy which nothing else can supply. And it will impel him onward and upward, and sustain him in a sphere of effort which selfishness can never reach. While the merely selfish man, who values and pursues his profession only for the dollars and cents it brings him, will rarely go further than he finds it profitable; the man who really esteems his profession will cultivate it for its own sake. Hence, no ordinary measure of attainment will ever satisfy him. He will desire, also, to extend its boundaries, and render it worthy of a still higher place in the public regard. His ambition will be to leave his profession more improved and more elevated than he found it. In addition, then, to those inferior motives which operate upon the merely business man, he who loves his profession has stronger and loftier impulses, which will be certain to render him far more accomplished, and, consequently, far more worthy of public confidence.

But this is not all. A mere regard to one's individual interests, that is, in one word—selfishness, is sure to belittle the man—it will render him a professional dwarf. But where a cordial regard for his profession is the animating and controling principle, it cannot fail to inspire the heart with refined, generous, noble sentiments, and thus impress true professional dignity upon the whole man.

II. *Always cherish feelings of respect for your fellow practitioners.*

"None of us liveth to himself." This is not more the teaching of holy writ, than a lesson of sound experience. The character and welfare of all the members of a profession are, to a great extent, bound up in the same bundle. If "one member suffer, all the members suffer with it, or one member be honored, all the members rejoice with it." Let but the profession be elevated and respected, and each individual shares in the general esteem. That is a miserable short-sighted policy which seeks to exalt itself upon the ruin of other men's reputations. I knew a man once of more than ordinary abilities :—as a student, his opportunities had been most ample ; he entered upon a medical career under highly propitious circumstances ; his energy was great, his industry untiring, his ambition unbounded. He had a liberal spirit, also, in respect to the learning of the profession, and to literature, and science generally. He spared no expense for books, apparatus, and whatever else was necessary to high attainment. But he did little good, and much evil, in the profession ; he lived unhappily in it, and, after a life of study, toil, and profuse expenditure, utterly failed of reaching a true renown. And to what was his disappointment owing? Not to a want of talents, or learning, or industry, or success as a practitioner, or to that low, contracted spirit, which denies to itself the means of professional improvement—his library was immense ; he gathered about himself every advantage of this kind which wealth and the most inordinate ambition could procure or desire. What then was the fatal cause of his disappointment? It was simply this—he did not cherish a feeling of respect for his fellow practitioners. This was the worm which caused the goodly gourd to wither over his head. He took little pains to conciliate the kind feelings of his brethren. He delighted to dwell upon their imperfections. He blazoned their faults. Indeed, nothing seemed to give him more pleasure in the professor's chair, than to expose the mistakes, or pour contemptuous sarcasm upon the character or course of some less favored member of the profession. As a matter of course, he was perpetually involved in heart-burnings and contests. Almost every one had some grievance to complain of against him. He soon became the Ishmael of the profession, "his hand against every man and every man's hand against him." Is it wonderful then, that notwithstanding all his advantages and opportunities, he utterly failed—became a splendid bankrupt in reputation? What can one man do against a whole profession?—and especially when his own selfish contemptuous treatment of them, has provoked their righteous indignation against him?

But if the ill will of a profession is so overwhelming an evil; one against which no talents, no attainments, no advantages, however great, can enable a man to bear up, how desirable, how full of strength and encouragement, on the other hand, are the respect and good will of the profession? These will open the way, and smooth its ascent, even to the most humble. Though a man's abilities be but moderate, yet in the possession of the respect and good will of his brethren, he has the surest means and the best guaranty of professional success. And when reputation does come, no breath of envy will ever tarnish his crown. All will delight to honor him.

But how may a medical man—in whatever department he may labor—secure to himself this high advantage, this most gratifying of all attainments, a profession's respect and good will? There is only one way, but that is a sure one—let him cherish a feeling of respect for his fellow practitioners. This will lead him always to regard their rights as sacred; this will render him at all times sincerely courteous, even to the most humble of his brethren.

III. *Ever cultivate the spirit of benevolence.*

There cannot be a brighter ornament in the character of a medical man, than benevolence. Without it, he may have the most vigorous intellect, and the greatest amount of general scholarship, and professional learning and practical skill, still his character will be miserably imperfect. No one can love him. None will freely approach him. Necessity may indeed compel some to seek his aid, but they will feel little gratitude for it, and retain no pleasant recollections of him.

A kindly spirit and gentle manners will wonderfully mitigate the sufferings of a patient, even where your skill fails to give relief. And hundreds under the influence of a sympathizing, winning deportment, may be induced to submit to tedious courses of treatment, and even painful operations, when nothing else could subdue. And thus a vast amount of human ills may be removed, which would otherwise be irremediable. It is in this way that a benevolent temper and its natural and usual accompaniment, gentle manners, greatly add to the effectiveness of the medical practitioner. Hence, one so qualified will win confidence and ensure success, where others of much larger professional learning and skill may utterly fail.

Is not the most common commendation which persons will give of their family physician, and their reason for preferring him, just this virtue of benevolence? "His manners," they will say, "are so kind and pleasant—he seems to have so much feeling for you!" And all this is perfectly natural and reasonable. Every one, and especially when suffering, can judge of the kindness of a professional man, and feel it too; but mere medical attainments must ever, to a great extent, necessarily remain occult to the uninitiated. None but professional men can properly estimate each other. We see the proof of this continually, in the fact that it is personal manners—not vigor of intellect, not great professional attainments—it is personal manners that usually win a man's way to success and distinction. Who of us that have reached middle age, have not often looked with equal disappointment and surprise upon the different conditions of our fellow students, after the lapse of twenty or thirty years from the day of our separation at commencement? Was it not emphatically true of them—"the race is not to the swift, nor the battle to the strong?" Those who gave the highest promise, as students, have often turned out the most signal failures; while many, who for the slenderness of their capacity, were the butts of their class, have, in many instances, attained no small measure of success, and at times acquired even an enviable reputation. If such cases be carefully scrutinized, it will be found, we are persuaded, that it was mainly difference of manners which wrought out their different destinies. The medical man of ordinary, or even less than ordinary intellect, but of kindly manners, will be almost certain to outstrip, in his professional career, his fellow student of an opposite character. And it is right—it is right, that it should be so, for we cannot help the feebleness of our minds, but we are to blame for the absence of a benevolent spirit and kind manners.

There is, however, another view of this point which it would be unpardonable, on the present occasion, to overlook. A benevolent spirit and kind manners are important, not merely as a means of soothing suffering and winning success. With these ends in view, a medical man may cultivate such a spirit and such manners—or rather the appearance of them—from the most selfish motives. But true benevolence, and true kindness will always be valuable in such a world as ours, from other considerations. "The poor shall never cease out of the land." This is the decree of the great disposer of human destiny, "the High and Holy One, who inhabitest eternity, and doeth according to his will in the army of heaven and among the inhabitants of the earth." "The poor," declares the meek and merciful Saviour, "always ye have with you." Who has not admired that noble saying of Boerhaave?—"The poor are my best patients, for God is their paymaster." And what can now add so amaranthine a lustre to the character of Rush, as this simple testimony of his biographer?—"The poor were the objects of his peculiar care. In the latter and more prosperous years of his life, one-seventh of his income was expended upon the children of affliction and want. The last expression which fell from his lips was an injunction to his son—"Be indulgent to the poor."

What was so tenderly said by the poet of one medical practitioner, we are verily persuaded, is true of the great body of the profession in every age:

> **"In misery's darkest cavern known,**
> **His useful care was ever nigh,**
> **Where hopeless anguish poured his groan,**
> **And lonely want retired to die."**
>
> *Dr. S. Johnson's Epitaph on Dr. Levet.*

Of all men engaged in secular pursuits, the medical practitioner has the largest sphere of benevolence open before him; and not only so, but with smaller means he can do a greater amount of good than others. Look, for example, at that poor man—he is suffering under a painful disease, which utterly disqualifies him for his daily labor. His family are in great need, and must every day sink into a lower depth of poverty and wretchedness so long as he, who is their only support, is unable to exert himself. Now, to provide for the wants of this family is all that non-professional benevolence can do, and that would be, perhaps, a long continued and very onerous service of charity. And yet do its utmost, such charity leaves the greatest sufferer unrelieved, while it places his family under a painful, and, too often, an injurious sense of obligation. But the practitioner of the healing art can frequently, at a very trifling expense, and in a very short time, remove the poor man's disease, relieve him of all his sufferings, restore him to his capacities for exertion, and thus bring plenty and joy to his habitation. For all this good, the poor man will have but very little—oftentimes (such is the benevolence of physicians) nothing at all to pay. And his family, instead of being in the humiliating and generally vitiating state of pauperism—the only state in which an ordinary benevolence usually can place them—his family we say, will be restored to a happy, virtuous independence. Such is the power of medical benevolence. Such are the triumphs it achieves.

And in justice to the profession, it ought to be added, that there is no class that exhibits a more benevolent spirit, or does more to relieve the sufferings of humanity. This is so remarkably the fact, that few things disappoint or shock us more than the sight of an unsympathising, hard-hearted practitioner of the healing art, in any of its departments.

But if the profession you have chosen is so important in its objects, and demands such continued and strenuous intellectual and moral effort, what can sustain you in your career? What supply the needed motives to such study, toil, self-sacrifice, and benevolent effort? I can point you to only one source of motives, but that, thank heaven, is all-sufficient. It is christian principle. Only this can give you such command of your intellect, such control over your heart as will enable you to fulfil your high vocation.

To the medical practitioner, therefore, who would study to show himself approved—who would labor and not faint—who would persevere in well-doing—I would say, go my friend, go in faith to the great physician of souls—the Lord Jesus Christ. Learn in his school—imbibe his spirit. Contemplate his condescending kindness, his unwearied labors, his atoning death. With a heart enlightened, and warmed, and melted, and elevated, in view of his redeeming love, you will find it, by a happy experience, not a task, but a delightful privilege to minister to the afflicted. Yes, my friend, true christian piety alone can sustain and carry you forward in such a career of professional labor and benevolence, as will, in this life, call down upon your head the blessings of multitudes ready to perish; and prepare you—when all this world's prospects have faded upon your sight, and its last sounds have died upon your ears—for a higher career of usefulness and of blessedness!

